# Human-Animal Co-Sleeping: An Actigraphy-Based Assessment of Dogs’ Impacts on Women’s Nighttime Movements

**DOI:** 10.3390/ani10020278

**Published:** 2020-02-11

**Authors:** Christy L. Hoffman, Matthew Browne, Bradley P. Smith

**Affiliations:** 1 Department of Animal Behavior, Ecology, and Conservation, Canisius College, Buffalo, 14208 NY, USA; 2 School of Health, Medical and Applied Sciences, Central Queensland University, Bundaberg 4670, Australia; m.browne@cqu.edu.au; 3 Appleton Institute for Behavioural Science, School of Health, Medical and Applied Sciences, Central Queensland University, Adelaide 5000, Australia; b.p.smith@cqu.edu.au

**Keywords:** actigraphy, bedsharing, co-sleeping, dogs, human–animal interaction, pets, sleep

## Abstract

**Simple Summary:**

Humans commonly share their beds with companion animals, yet little is known regarding how pets impact sleep. In survey-based studies, pet owners report that their dogs favorably affect sleep quality, but prior actigraphy-based studies of human-dog co-sleeping have concluded the practice can lead to sleep arousals and disturbances and, thus, reduce sleep efficiency (i.e., the ratio of time spent asleep in a night as compared to the time spent in bed). We examined actigraphy data from women and dogs and sleep diary data to investigate the apparent disconnect between objective and subjective reports regarding dogs’ effects on sleep. We also analyzed data minute-by-minute to assess whether dog movement impacted the likelihood the human would transition from an inactive to active state. We found associations between human and dog movement over sleep periods and that dogs influenced human movement more than humans influenced dog movement. Humans were largely unaware of their dog’s nighttime movements, and they rarely reported that their dog awakened them during the night. Additional research on more diverse samples and studies that use polysomnography and behavioral observations are necessary for developing a better understanding of how pets affect the quality of human sleep.

**Abstract:**

Humans regularly enter into co-sleeping arrangements with human and non-human partners. Studies of adults who co-sleep report that co-sleeping can impact sleep quality, particularly for women. Although dog owners often choose to bedshare with their dogs, we know relatively little about the nature of these relationships, nor the extent to which co-sleeping might interfere with sleep quality or quantity. In an effort to rectify this, we selected a sample of 12 adult female human (*M* = 50.8 years) and dog dyads, and monitored their activity using actigraphy. We collected movement data in one-minute epochs for each sleep period for an average of 10 nights per participant. This resulted in 124 nights of data, covering 54,533 observations (*M* = 7.3 hours per night). In addition, we collected subjective sleep diary data from human participants. We found a significant positive relationship between human and dog movement over sleep periods, with dogs influencing human movement more than humans influenced dog movement. Dog movement accompanied approximately 50% of human movement observations, and dog movement tripled the likelihood of the human transitioning from a non-moving state to a moving state. Nevertheless, humans rarely reported that their dog disrupted their sleep. We encourage the continued exploration of human-animal co-sleeping in all its facets and provide recommendations for future research in this area.

## 1. Introduction

Co-sleeping is a common human behavior that reflects a variety of cultural, social, and psychological phenomena [1]. This appears consistent, regardless of whether the sleeping partner is a human adult, human child, or non-human animal [2]. The practice of co-sleeping (i.e., sharing one’s bed or bedroom) with animals is a long-standing one in both traditional and modern social contexts, with contemporary dog and cat owners in various parts of the world commonly sharing their sleeping space with their pet [3,4]. The presence of pets in the bedroom is not surprising, given that 37% of households in the United States include a dog, 30% include a cat, and 63% of pet-owning households consider their pets to be family members [5].

The relationship between humans and their companion animals is an important one, with numerous health-related and psychological benefits having been linked to pet ownership [6]. Factors that are thought to moderate the relationship between pet ownership and health have commonly focused on ways in which pets, particularly dogs, positively impact physical activity and psychological well-being [7,8,9,10]. Nevertheless, the presence of dogs might also influence the quality of human sleep--a crucial activity required for a wide spectrum of physiological and mental processes [11]. Indeed, dogs may have initially gained acceptance in human settlements thousands of years ago because they help to keep humans warm during cold nights [12], and because of their tendency to bark and deter potential threats [13,14]. Thus, in early human societies, dogs likely increased the amount of uninterrupted time that humans could devote to sleep during nighttime hours.

Bedsharing, whether it be with an adult partner, child, or pet, is a co-regulated activity [15], meaning that there is some degree of synchrony of physiological processes between individuals who share a bed. The importance of the practice should not be understated. For example, humans who sleep together in the same bed share approximately one-third of their nocturnal awakenings [16]. Further, individuals who sleep with a human partner turn out the lights earlier, and women fall asleep faster when their human partner is present [16]. However, there are many physiological (e.g., circadian rhythms, monophasic/polyphasic sleep) and behavioral (e.g., toilet breaks, noise, and movement) factors that can lead to sleep disruption [1,17]. Actigraphic measures of sleep efficiency (i.e., the ratio of time spent asleep in a night as compared to the time spent in bed) and self-report measures of sleep quality suggest that women are more adversely affected by bed-sharing with adult partners than men, possibly because, across human history, bedsharing has typically occurred between mothers and their highly dependent infants [18]. Thus, despite the many benefits of couple (human-human) co-sleeping, its effects on sleep quality and physical and mental health are not entirely positive [1].

Dog owners frequently report that the psychological benefits of co-sleeping outweigh the sleep disturbances and dog behavioral problems that might also arise from it [19]. The same is true for human-human co-sleepers [1]. Additionally, like their human-human co-sleeper counterparts, many pet owner co-sleepers believe their pets help them relax and feel more secure, thereby enabling them to sleep better [3,19,20,21]. Co-sleeping is also associated with stronger owner attachment to the dog [22]. However, people who share their bed with their pets take longer to fall asleep, experience more sleep disturbances, and are more likely to wake up tired [19]. They also often fail to recognize that their sleep might be impacted [17], and may overlook the risks that are associated with sharing their bed with their dog. For example, bedsharing with a pet increases the risk of zoonotic disease transmission [23], and potentially interferes with human-human relationships [4]. 

While it is common for humans to share their homes and beds with pets, few studies have focused on human-animal co-sleeping, and those that have heavily relied upon self-report data [3,19,20,21,24]. Only two studies have utilized actigraphy to objectively examine the impact of co-sleeping on human sleep and pet sleep [17,25]. Data from both studies indicate that dogs do reduce their owner’s sleep efficiency. However, unfortunately, both suffer important limitations. For instance, although the actigraphy study that was conducted by Smith et al. [17] succeeded in highlighting the diversity of sleeping arrangements and reasons why dog owners start and continue to co-sleep, it only included a small ad-hoc sample of five females who each provided seven nights of data. Furthermore, the study did not control for co-sleeping partners (human or non-human) or context (e.g., number of dogs, age, and occupation of human). While Patel et al. [25] had a large sample of participants and concluded that human sleep efficiency differed depending on where the dog slept (e.g., on the bed, on the bedroom floor), they did not control for human bed partners, and they only reported the average number of minutes that dogs and owners were sleeping or resting. That is, they did not examine minute-to-minute associations between dog and human movement to determine the frequencies at which dog movement preceded or coincided with human movement. Additionally, although Patel et al. collected participants’ reports regarding sleep quality, they did not examine them in relation to activity, as measured by the participants’ accelerometers. 

The relationships humans have with their companion animals have received considerable attention [26,27,28], and a great deal is known about human sleep more generally [11]. However, little is known about the interaction between the two—particularly the extent to which co-sleeping with a dog might disrupt the sleep of the human. Furthermore, the few studies conducted to date have yielded inconsistent results. That is, the actigraphy-based findings indicate that dogs impair sleep, whereas self-report data indicate that owners believe their dogs have positive impacts on their sleep. Following up on findings from prior co-sleeping studies, we selected a sample of bedsharing adult human (female) and dog dyads and analyzed their movement while sleeping as well as their daily sleep diary reports. We expected that dogs’ nighttime movements would precipitate humans’ nighttime movements, but that dog owners would be unlikely to associate poor sleep quality with their dog’s nighttime activities, based on conclusions drawn from prior studies of co-sleeping between humans, as well as between humans and dogs.

## 2. Materials and Methods

### 2.1. Participants

We recruited female participants from a pool of individuals who had completed a survey for a related study of the perceived effects of pets on sleep (see Hoffman et al. [3]). The participants had to be adult women residing within 50 miles of Buffalo, NY, who reported routinely sleeping at night (i.e., they could not be shift workers); furthermore, they could not have any physical or psychological condition, or take any medication known to alter sleep patterns. In addition, participants had to have one dog and no other dogs or cats, and they could not share their bed with a human partner. Finally, individuals had to indicate their willingness to wear an accelerometer on their wrist, and allow their dog to wear one on their collar, for up to 14 days. We did not screen dogs for any existing medical conditions. 

Sixteen women and their dogs participated in this study between November 2016 and May 2017. All of the participants completed the study, and each received a $50 gift card upon returning study materials. Four participants did not share their bed with their dog for more than one full night during the data collection period, and so we excluded them from the dataset. As such, the analyses herein are restricted to the 12 women who shared their bed with their dog (and no other human or animal) during at least two full nights of the study. These 12 participants ranged in age from 26.3 years to 65.6 years (*M* = 50.8, *SD* = 12.0) and weighed between 50.4 and 111.1 kg (*M* = 74.8, *SD* = 15.9). The dogs were between one year and 13 years (*M* = 5.5, *SD* = 4.1) and weighed between 4.1 and 31.8 kg (*M* = 14.8, *SD* = 9.6). Nine dogs were male and three were female.

### 2.2. Procedures

**Figure 1:**
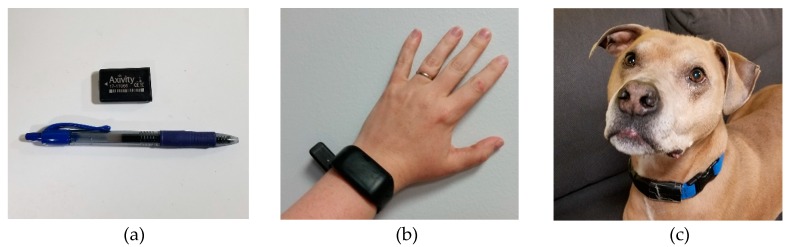
Study equipment. The figure on the left (**a**) shows the size of the Axivity AX3 in relation to an ink pen; the middle figure (**b**) shows how the Axivity AX3 was housed inside a wristband that human participants wore; and the figure on the right (**c**) shows the Axivity AX3 attached to the dog’s collar.

Each participant wore an Axivity AX3 accelerometer (Axivity, Newcastle upon Tyne, UK), which has previously been validated for detecting humans’ and dogs’ subtle movements during sleep periods and estimating human sleep efficiency [29,30]. This device is small and unobtrusive to the wearer (11g, 23 × 32.5 × 7.6 mm; Figure 1). For human participants, the AX3 device was secured inside the cavity of a skin-safe, silicone wristband that was worn on the wrist of each participant’s non-dominant hand. The wristbands weighed 16g and they were cleaned with isopropyl alcohol before they were distributed to participants. A second AX3 device was affixed to a nylon dog collar that we provided. We asked that owners allow their dog to wear this collar in addition to any collar that their dog typically wears, and we taped over the collar’s D ring to ensure that owners did not attach a leash to it. We adjusted the collar so that it fit snugly, allowing only two or three fingers to fit between the dog’s neck and the collar. The owners could remove the accelerometer and collar from the dog when the dog was engaging in any activities during which wearing a collar was deemed unsafe. For example, some dogs attended dog daycares that did not allow dogs to wear collars due to safety concerns.

Acceleration was measured at a 100-Hz sampling rate, and the data were grouped into 60s epochs. The open source software Open Movement (Newcastle University, Newcastle, UK) that is compatible with the AX3 device provided readings of signal vector magnitude (SVM) displacement of three axes (anteroposterior, mediolateral, and vertical) for human participants. The dog data were uploaded to, and processed by, the web-based ActivityScope program (VetSens, Newcastle, UK). ActivityScope provided the composite vector magnitude (VM3) for each 60s epoch. 

A member of the study team delivered the AX3s to participants’ homes, and participants and their dogs wore the accelerometers for 14 consecutive days. In addition to wearing the AX3, study participants completed a brief survey each morning upon waking. The survey included questions from the Consensus Sleep Diary [31], which is a standardized, commonly used tool that sleep researchers developed for collecting individuals’ daily assessments of their previous night’s sleep quality (e.g., “How many times did you wake up, not counting your final awakening?”). The survey also asked participants to note any sleep disturbances that were caused by their dog or other humans in the home. A study team member retrieved the devices and sleep diary reports at the conclusion of each participant’s study period. 

Study approval was obtained from both the Institutional Animal Care and Use Committee (2016 0413 205) and the Institutional Review Board (IRB 2015-16#86) at Canisius College.

### 2.3. Thresholding

According to the 2011 Compendium of Physical Activities [32], energy expenditure during sleep is equivalent to 0.95 metabolic equivalent of task (MET), and the estimates for lying in bed awake range from 1.0 METs to 1.3 METs. We set the threshold for detecting human nighttime activity at 1.1 METs, which corresponded to 91 SVM_gs_ (g·min), and is well below the 217 SVM_gs_ (1.5 METs) threshold that divides sedentary activities from light physical activities, such as standing or walking slowly [33]. 

We reviewed video data originally collected for a study validating the use of actigraphy to identify periods of dog resting behavior to determine the threshold for identifying dogs’ nighttime movements (see Ladha and Hoffman [30]). Dogs’ movements below 150 counts per minute (cpm) were difficult to detect while reviewing the videos in real time; movements between 150 cpm and 400 cpm corresponded to dogs making subtle, yet visible, adjustments to the positioning of their head or legs. A complete shift of the dog’s head and legs while the dog remained lying down (e.g., dog moves from laying prone with head aligned with body to lifting head and twisting the head and body into a circular shape) consistently registered above 400 cpm. Activities, such as moving from lying down to sitting or standing typically exceeded 800 cpm, and scratching or licking a body part for several seconds registered over 1000 cpm. Based on this information, we selected 400 cpm as the threshold for detecting dog nighttime activity, which falls below the 1352 cpm threshold that divides sedentary activities from light to moderate activities [34]. 

### 2.4. Data Screening

Participants completed the sleep diary each morning, but the data were only analyzed for those nights during which the dog remained on the bed the full night. We categorized the dog as having spent the full night in bed if the participant indicated that the dog was in bed for a period that met or exceeded the amount of time the participant reported sleeping, or if the participant’s estimate of the time the dog was in bed fell short of their sleep time estimate by 15 or fewer minutes.

### 2.5. Data Analysis

We conducted analyses in the R statistical programming environment [35]. Human movement recordings were thresholded at 91 SVM_gs_ and dog movement was thresholded at 400 cpm to create binary movement/no movement state variables (see Thresholding section above). We analyzed dog and human activity while using classical non-parametric tests, including the Wilcoxon rank-sum test and a chi-square test. We also used continuous time Multi-State Markov Models (MSM), which were run using the msm package [36]. Similar results could also be achieved by employing multinomial logistic regression because the data were gathered at discrete and fixed intervals. However, MSMs provide a convenient framework by which to evaluate whether preceding dog movement increased the probability of a person transitioning from a non-moving to a moving state, and vice versa. A sleep period index, varying over dyad and night, was also included as a grouping variable. We created lagged (t - 1) versions of dog and human recordings to address specific questions. We employed the lme4 package [37] to implement a linear mixed effects (LME) model, which took the potential heterogeneity of effects over dyads into account. In predicting subjective sleep quality, the LME model predicted a five-point Likert scale subjective rating of nightly sleep quality, while using the proportion of time the dog spent moving during the night as a fixed effect. A random intercept for dyad was included in the model to take into account individual differences.

## 3. Results

After screening, a total of 124 sleep periods were available for analysis, comprising a total of 54,533 observations, which corresponds to an average 7.3 hour (*SD* = 1.13) monitoring period per night. The participants provided, on average, 10.3 nights of data (*SD* = 3.39). Supplementary Figures S1 and S2 illustrate the density distribution of raw AX3 measurements separately for each dyad and period for dogs and humans, respectively. Averaged over sleep periods, humans spent an average of 4.5% (*SD* = 2.67) of a sleep period in a moving state, and dogs spent an average of 12.4% (*SD* = 6.90) of a sleep period in a moving state. The total human moving time per period was approximately normally distributed over nights, whilst dog moving time was subject to mild positive skew. 

There was a significant positive relationship between human and dog movement over sleep periods, *r* = 0.46, *t* (122) = 5.89, *p* < 0.001. Thus, sleep efficiency was lower for humans during sleep periods in which dogs were more active. We fitted a basic LME model predicting total human movement by total dog movement (expressed as a proportion) over nights, incorporating a random intercept and slope for dyad to check whether this relationship persisted after controlling for heterogeneity over dyads. A similar significant positive relationship was observed, *B* = 0.29 (*SE* = 0.043), *t* = 6.88, *p* < 0.01.

**Figure 2:**
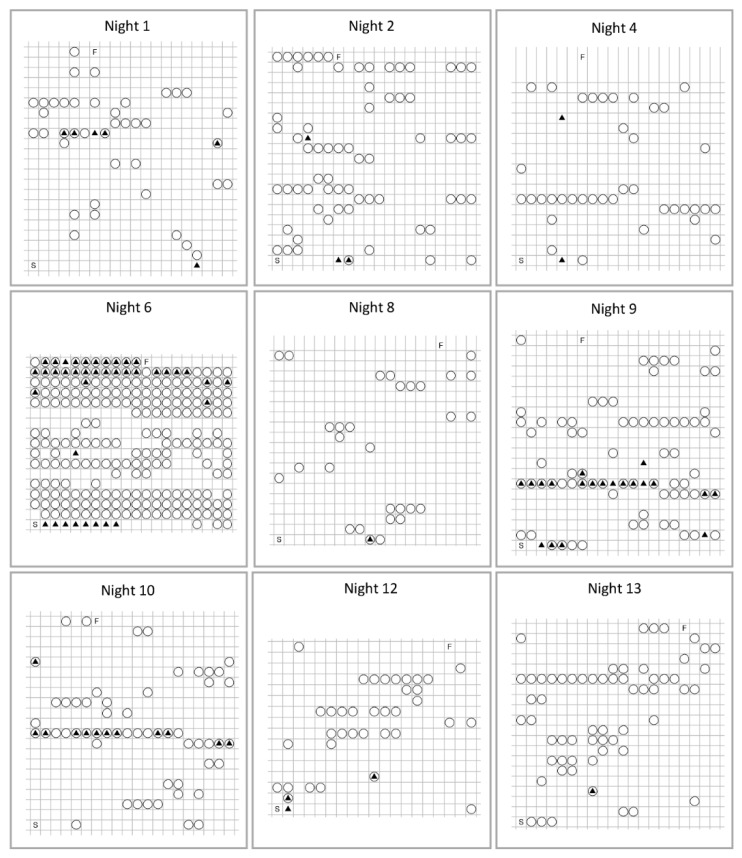
The movement states for one human-dog dyad across a selection of sleep periods (nights). These illustrations reflect a variety of movement patterns by dog and human when co-sleeping. For example, dog and human sleeping together with little movement (night 8); bouts of high level of dog movement with little human movement (nights 2, 4, 12, 13); and, high levels of dog and human movement (nights 6, 9). Each cell represents a 1-minute epoch, and should be read from bottom left of matrix to top right. Beginning of period indicated by S (start) and completion of sleep period indicated by F (finish). ▢ = no human or dog movement; ▲ = human movement only; ◯ = dog movement only; ◯▲ = both human and dog movement.

Dog and human movement were positively related at a minute-by-minute level. Table 1 cross-tabulates human and dog movement. It illustrates that dog movement accompanied 50% of human movement observations, whereas the baseline rates of movement were 4.5% for humans and 12.4% for dogs. This rate of positive co-occurrence was significant, *X*^2^(1) = 3295.2, *p* < 0.01. Figure 2 presents illustrative examples of dog and human movement within one dyad across nine nights. These examples highlight the diversity of movement frequencies and co-occurrences between human and dog that can occur within a dyad across nights.

Our Markov State Model (MSM) focused on assessing the degree to which movement by dogs and humans at a previous time step (t − 1) was related to subsequent movement of the bed partner at time (t). We first treated movement as a 2×2 dyadic state, being defined in terms of whether or not either or both entities were in a movement state. Table 2 details the number of state transitions observed in the total dataset, and the probability of each state transition. When only the dog was moving, the probability of the human transitioning to a movement state was 0.053 + 0.021 = 0.074. When the dog was not moving, the probability of the human transitioning was 0.006 + 0.015 = 0.021. Thus, the raw increased odds of a human transitioning to a movement state during dog movement was 0.074/0.021 = 3.52 times. When only the human was moving, the probability of the dog transitioning to a movement state was 0.086 + 0.043 = 0.129, and the probability of the dog transitioning was 0.006 + 0.057 = 0.063 when the human was not moving. The raw increased odds of a dog transitioning to movement during human movement was 0.129/0.063 = 2.04 times.

The increased hazard of a human commencing movement was formally assessed using a simple binary MSM, in which only the two human states (i.e., movement, no movement) were considered, with dog movement at (t − 1) included as a covariate. A second covariate, proportion of the sleep period (ranging from zero to one), was also included to assess whether the effects varied with respect to proportion of the sleep period that had passed. Table 3 summarizes the increased hazard of human state transitions as a function of dog movement and proportion of night that had passed.

Dog movement increased the likelihood of the human transitioning from a non-moving state to a moving state by about three times, and decreased the likelihood of the person transitioning from a moving to a non-moving state, as shown in Table 3. Humans were also significantly more likely to transition between non-moving and moving states as the sleep period progressed. However, this effect was substantially smaller than the effect of dog movement. We found no evidence of an interaction effect between preceding dog movement and the proportion of time elapsed through the sleep period.

Finally, we tested whether the duration of human movement periods was related to the presence of dog movement in the minute prior to onset. We calculated the run-length consecutive sequences of all human movement states, and then conducted a Wilcoxon rank-sum test to compare those that were preceded by dog movement and those that were not. The average duration of human movement periods that were preceded by dog movement was 1.92 minutes (415 cases), as compared to 1.49 minutes (1015 cases) when not being preceded by dog movement. This difference was significant, *W* = 235635, *p* < 0.01.

### Subjective versus Objective Sleep

Subjective evaluations of sleep quality that participants reported in the sleep diary were significantly related to the proportion of time that the actigraphy data indicated the human spent in a moving state, *r* = 0.33, *p* < 0.01. We employed an LME model with a random intercept for dyad predicting subjective ratings of human sleep quality based on the proportion of time the dog was active and found a significant effect, *B* = −3.51 (*SE* = 1.18), *t*_(121.5)_ = −2.98, *p* < 0.01. The addition of a random slope for dog activity did not improve model fit, Δχ^2^ (2) = 1.60, *p* = 0.49. Thus, there was no evidence for heterogeneity of effect over dyads. Of the total variance in subjective sleep ratings, 7.1% was explained by differing levels of nightly canine activity, 31.8% by between-dyad effects, and the residual 61.1% being unexplained within-dyad variance. However, caution should be exercised in interpreting these variance components, due to the ordinal (1–5) scale of the subjective sleep ratings. Although the total proportion of time that dogs spent in a moving state was associated with poorer subjective assessments of sleep quality, participants only recollected being awakened by their dog on 22 of the 124 sleep periods examined. Furthermore, six of those 22 sleep periods were reported by one individual. Additionally, three of the 12 participants indicated that their dog never woke them during the study period.

## 4. Discussion

Co-sleeping dogs’ and humans’ movements during sleep periods were moderately correlated. That is, heightened dog movements were positively associated with heightened human movements. This finding is consistent with the negative association between dog activity and human sleep efficiency that prior studies have reported [17,25]. However, this association alone does not allow for us to infer a causal relationship between dog activity and human sleep disturbance. Human activity, dog activity, or any number of external variables could have driven the relationship that we observed between dog and human movement. For instance, although we restricted our analyses to data that were collected from women who shared their bed with one dog and no other bed partners (human or nonhuman), some participants had other humans in their household, whose actions may have contributed to sleep disturbances. Furthermore, some participants and their dogs may have been affected by events that we were not aware of, such as noises coming from outside their home, particularly if they lived in a high traffic area.

We examined how often dogs and humans were in an active state and whether the movement of one bed partner had any relationship to the movement of the other to further investigate the relationship between dog and human nighttime activity. Dogs were in an active state nearly three times as often as their human bed partners, which is to be expected given differences in dogs’ and humans’ sleep-wake cycles [38]. Half of all human movements co-occurred with dog movements, whereas only 18% of all dog movements co-occurred with human movements. Humans were three times more likely to transition to a movement state during the minute that followed dog movement when compared to times when the dog was not moving. With dogs influencing human movement more than humans influenced dog movement, it is clear that the temporal relationship is not symmetrical. This finding confirms those of Smith et al. [17]. However, bedsharing with a dog for many human-dog dyads represents a co-regulated activity despite co-sleeping humans’ and dogs’ impacts on the other’s movements not being as equitable as has been reported for human dyads [15]. This further supports the notion that human-animal co-sleeping arrangements represent genuine forms of co-sleeping that are often overlooked [2,4].

Given that, on average, dogs spent 12.4% of the night moving and humans only 4.5% of the night, the majority of dogs’ nighttime movements do not appear to adversely affect their human bed partners. However, in instances where human movement periods were immediately preceded by dog movement, those human movement periods lasted, on average, 26 seconds longer than those that were not preceded by dog movement. This suggests that dog movements are more disruptive than are disturbances unrelated to dog movement. Further investigation is needed to determine whether this 26-second difference has a meaningful impact on sleep quality.

While our findings indicate that dogs spend more of the night moving than their human bed partners and that human and dog movements are temporally linked, the actigraphy data did not confirm whether the participants were actually awake during movement periods. We carefully selected human and dog activity thresholds to identify periods of movement, yet not all movements above the threshold necessarily lead to an arousal or awakening. Polysomnography (PSG), which is the “gold standard” for measuring sleep in clinical settings, would provide more accurate information regarding participants’ sleep stages and periods of sleep disturbance, but the low-cost, non-invasive nature of actigraphy makes using it more feasible. Non-invasive PSG methods for measuring canine sleep are now readily available [39], but they still require behaviorally sound dogs that will tolerate sleeping with electrodes that are attached to their head. Fortunately, although actigraphy is not as reliable as PSG for detecting epochs when individuals are awake, actigraphy-based assessments of total overnight sleep time are strongly correlated with PSG [40,41,42]. Behavioral observations of video captured during sleep may also reveal more about the nature of human-dog co-sleeping and its impacts on both the human and dog. For instance, whereas our study relied on owners’ estimates of the amount of time dogs spent on the bed while the owners slept, video would provide more precise measures of the dog’s time on the bed.

Despite limitations that are inherent in actigraphy-based assessments of sleep, our use of both subjective and objective measures provided insight into why findings that are based on subjective reports of co-sleeping with dogs have tended to be positive [3,19,20,21], whereas objective measures have suggested that dogs are creating sleep arousals or disruptions [17,25]. We found that how well participants rated each night’s sleep was negatively associated with both human and dog movement during that night. That is, nights with more dog movement were associated with more human movement and poorer perceived sleep quality. Nevertheless, participants only recollected being disturbed by their dog on 22 of 124 nights. It seems that humans are not consciously associating their nights of poor sleep with their dog’s nighttime activities, given how little participants recalled dog-related sleep disruptions in relation to how much dog movement we observed across nights. Studies of adult humans who share a bed have yielded similar findings. Humans commonly indicate their sleep quality is better when their partner is present [16,43], yet actigraphy data reveal that they move less on the nights they sleep alone [16]. This discrepancy suggests that, despite the disturbances that bed partners create, they may be fulfilling a psychological need for feeling safe and secure during sleep periods [44]. It is important to add that human movement caused by dogs or other bed partners does not necessarily mean that sleep is being disrupted in a meaningful way [45]. For example, disruptions of short durations may not lead to daytime impairments. The negative impacts of co-sleeping may be the exception rather than the rule, given that the nature of human-dog co-sleeping arrangements are diverse and the practice so common. 

### Future Directions

While this study has provided novel insights into the effects of co-sleeping on dogs’ and women’s movement patterns, larger samples are needed to assess how human-dog co-sleeping impacts human health and daily functioning, and how the characteristics of both humans and dogs may moderate these impacts. Dog-related factors that might influence co-sleeping outcomes include dog age, size, and length of time in the household. A dog’s health status might be yet another factor, particularly if the dog’s condition leads to frequent scratching, coughing, or snoring. In addition, the dog’s training and behavioral issues, the owner’s reason for acquiring the dog, the owner’s attachment to the dog, owner gender, and owner health are factors that might affect outcomes. Experimental studies that are based upon within-subjects comparisons that, ideally, utilize behavioral observations and PSG are needed to test whether individuals sleep better or worse when their dog is in the bed. Future studies could also match individuals who sleep with their dog in their bed or bedroom with dog owners who do not. Such studies could directly measure whether the apparent costs of co-sleeping that actigraphy-based studies have identified are consistent across individuals and outweigh the psychological benefits of co-sleeping that owners have reported [3,19,20,21]. 

The human-pet co-sleeping studies published to date have primarily focused on adult women and their dogs. Follow-up work is needed to determine whether there are similar associations in nighttime activity between co-sleeping dogs and adult men or children, especially since human co-sleeping studies indicate that bed partners differently impact adult female, adult male, and child sleep [18,46]. Furthermore, it is important to evaluate objectively the effects of cats on human sleep, as cats commonly co-sleep with their owners [3]. It is hypothesized that cats create more nighttime disruptions than dogs since dogs tend to synchronize their sleep patterns with their humans [47], and their major sleep period more closely aligns with humans’ than do cats’ [48,49]. Indeed, a recent study found that bedsharing dogs stay in bed for all or most of the night, whereas bedsharing cats rarely stay in the bed through the night [3].

Actigraphy-based studies might also assess the ways that modern human lifestyles impact dogs’ resting behaviors. A recent actigraphy-based study reported that dogs residing in an animal shelter were more active than owned dogs throughout most of the day, including during the dogs’ five consecutive hours of least activity [50]. Additional studies might examine anthropogenic factors impacting dog resting behavior by tracking this behavior under various in-home sleeping arrangements (e.g., in the bed, in the bedroom, outside the bedroom), or by comparing the resting behaviors of dogs kept in homes with dogs who roam freely.

## 5. Conclusions

Our findings, along with those from other recent studies [17,25], present strong indications that having a dog in the bed is correlated with an increase in human movement—and potential sleep disturbances and awakenings—during sleep periods. They highlight that despite the nature of the co-sleeping interactions being rather one-sided, with dogs much more likely to disturb the human’s sleep, co-sleeping with a dog appears to be a co-regulated activity. We also found that participants rarely recalled being awakened by their dog’s nighttime movements, confirming previous reports that humans may not be fully aware of the ways their dog’s movements are linked to their sleep quality [17]. We encourage further studies to determine whether the patterns that we identified in females are generalizable to wider populations, and whether dog-related disturbances meaningfully impact human health and daily functioning. Although co-sleeping with dogs has some adverse effects on sleep efficiency, the practice remains common. Decisions relating to co-sleeping are complex and, overall, the benefits of co-sleeping may outweigh any negatives. An exploration of human-animal co-sleeping can reveal a wealth of information regarding human behavior and the intersection between humans and animals. Therefore, we encourage the continued exploration of human-animal co-sleeping in all its facets.

## Figures and Tables

**Table 1:** Cross tabulation of simultaneous human and dog movement.

Dog Movement	Human Movement	
	No	Yes
No	46,532 (89.4%)	1,235 (50.3%)
Yes	5,554 (10.6%)	1,222 (49.7%)
TOTAL	52,076 (100%)	2,457 (100%)

**Table 2:** Transition probabilities for 2 × 2 state model.

FROM Prior (t − 1)	*N*	TO Current (t)			
		Both	Human Only	Dog Only	Neither
Both	1198	0.399	0.09	0.247	0.262
Human Only	1226	0.086	0.239	0.043	0.631
Dog Only	5512	0.053	0.021	0.457	0.467
Neither	46473	0.006	0.015	0.057	0.921

**Table 3:** Effects of dog movement and proportion of night passed on human transitions from a moving to a non-moving state.

Covariate	Human Transition	Odds Ratio	Confidence Interval (99%)	
			Lower	Upper
Dog Movement Present	No Move to Move	2.98	2.52	3.52
	Move to No Move	0.64	0.56	0.75
Proportion of Night Passed	No Move to Move	1.33	1.02	1.73

## Supplementary Materials

The following are available online at https://www.mdpi.com/2076-2615/10/2/278/s1, Figure S1: Histograms of human SVM measurements, by dyad and night, Figure S2: Histograms of dog VM3 measurements, by dyad and night.
