# Supplementary material for: Human-Animal Co-Sleeping: An Actigraphy-Based Assessment of Dogs’ Impacts on Women’s Nighttime Movements
Source: Animals (Basel). 2020 Feb 11;10(2):278. doi: 10.3390/ani10020278 (PMC7070703; doi:10.3390/ani10020278)

Article

Supplementary Files: Human-Animal Co-Sleeping: An Actigraphy-Based Assessment of Dogs' Impacts on Women's Nighttime Movements

Christy L. Hoffman [1,*], Matthew Browne [2], and Bradley P. Smith [3]

Figure S1. Histograms of human SVM measurements, by dyad and night. Both x and y axes are on the logarithmic scale. Plots are arranged with respect to night (horizontal) and dyad (vertical) index. The number of available observations (i.e. minutes during the sleep period) is annotated for each plot. Blank cells indicate nights when human and dog did not spend the full night in the same bed, the human and/or dog did not wear the accelerometer, or a second human was in the bed.

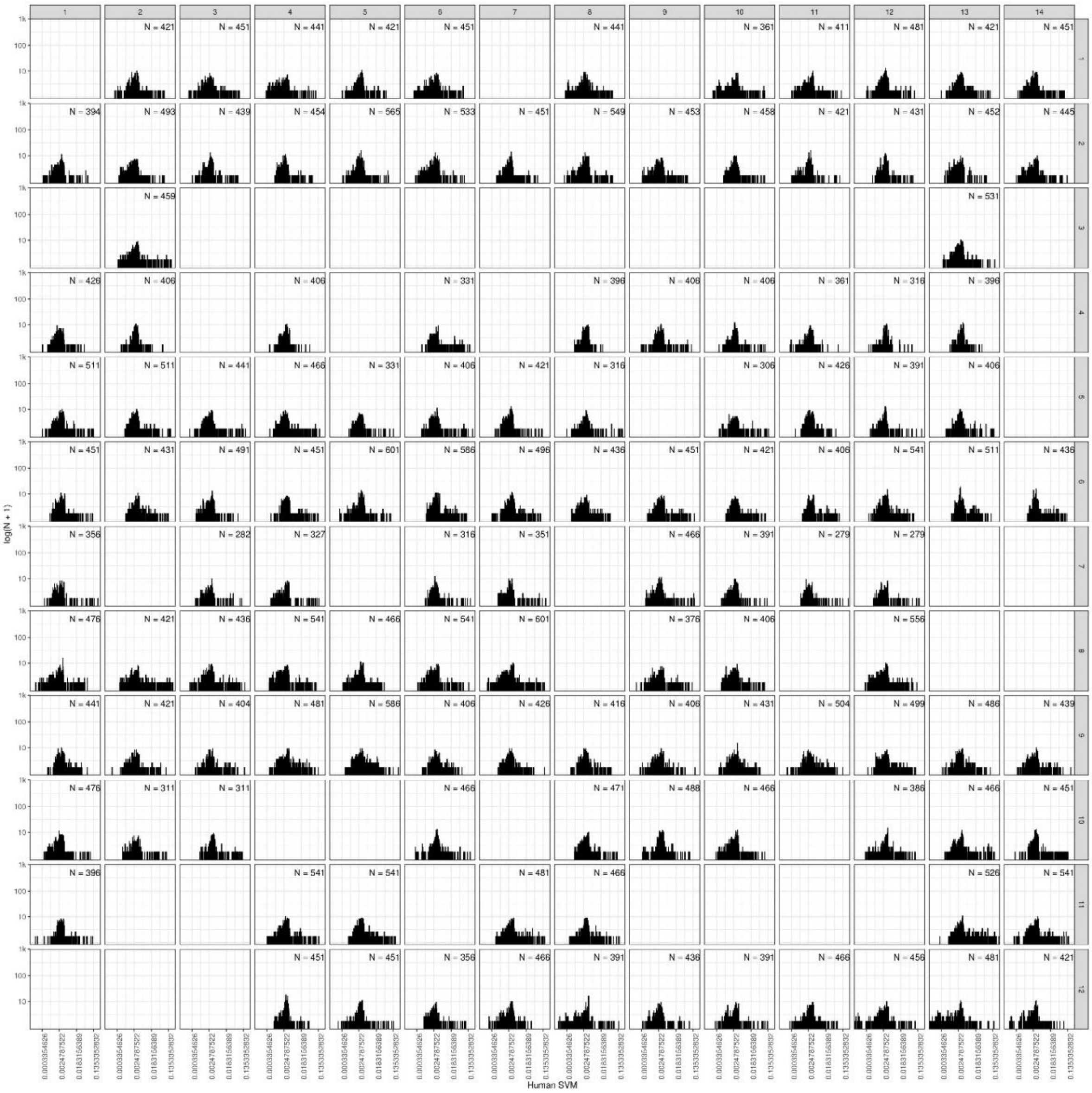

Figure S2. Histograms of dog VM3 measurements, by dyad and night. Both x and y axes are on the logarithmic scale. Plots are arranged with respect to night (horizontal) and dyad (vertical) index. The number of available observations (i.e. minutes during the sleep period) is annotated for each plot. Blank cells indicate nights when human and dog did not spend the full night in the same bed, the human and/or dog did not wear the accelerometer, or a second human was in the bed.

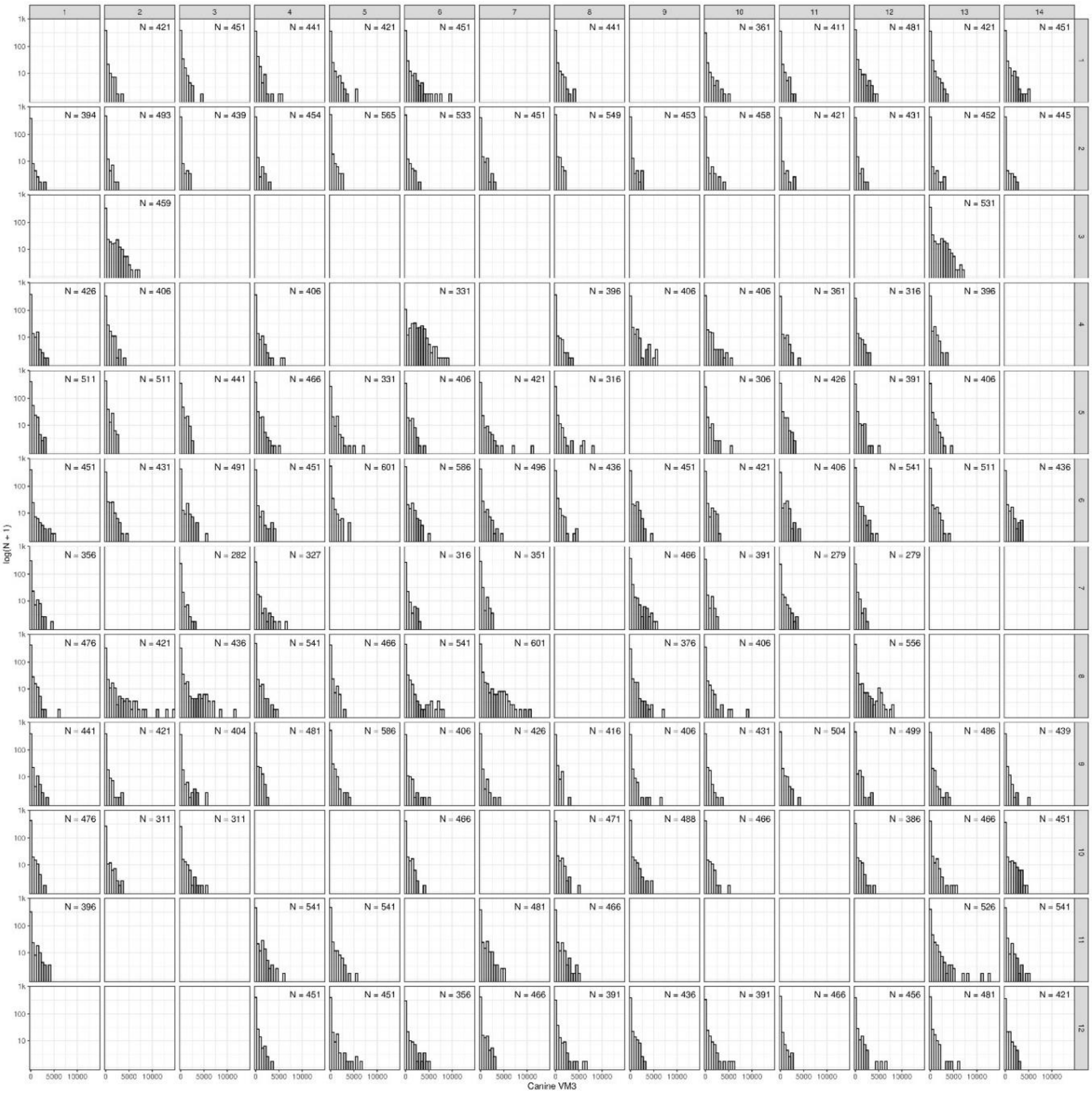

Supplement: Supplementary file 1 [file animals-10-00278-s001.pdf]
